# Functional traits composition predict macrophytes community productivity along a water depth gradient in a freshwater lake

**DOI:** 10.1002/ece3.1022

**Published:** 2014-03-26

**Authors:** Hui Fu, Jiayou Zhong, Guixiang Yuan, Leyi Ni, Ping Xie, Te Cao

**Affiliations:** 1 Jiangxi Provincial Key Laboratory of Water Resources and Environment of Poyang Lake, Jiangxi Institute of Water Sciences, NanchangChina; 2 Donghu Experimental Station of Lake Ecosystems, State Key Laboratory of Freshwater Ecology and Biotechnology, Institute of Hydrobiology, The Chinese Academy of SciencesWuhan, China

**Keywords:** Aquatic macrophytes, community productivity, community-weighted means, functional diversity, functional trait composition, water depth gradient

## Abstract

Functional trait composition of plant communities has been proposed as a helpful key for understanding the mechanisms of biodiversity effects on ecosystem functioning. In this study, we applied a step-wise modeling procedure to test the relative effects of taxonomic diversity, functional identity, and functional diversity on macrophytes community productivity along water depth gradient. We sampled 42 plots and 1513 individual plants and measured 16 functional traits and abundance of 17 macrophyte species. Results showed that there was a significant decrease in taxonomic diversity, functional identity (i.e., stem dry mass content, leaf [C] and leaf [N]), and functional diversity (i.e., floating leaf, mean Julian flowering date and rooting depth) with increasing water depth. For the multiple-trait functional diversity (FD) indices, functional richness decreased, while functional divergence increased with water depth gradient. Macrophyte community productivity was strongly determined by functional trait composition within community, but not significantly affected by taxonomic diversity. Community-weighted means (CWM) showed a two times higher explanatory power relative to FD indices in determining variations in community productivity. For nine of sixteen traits, CWM and FD showed significant correlations with community productivity, although the strength and direction of those relations depended on selected trait. Furthermore, functional composition in a community affected productivity through either additive or opposite effects of CWM and FD, depending on the particular traits being considered. Our results suggested both mechanisms of mass ratio and niche complementarity can operate simultaneously on variations in community productivity, and considering both CWM and FD would lead to a more profound understanding of traits–productivity relationships.

## Introduction

Experimental and field researches have documented a positive relationship between biodiversity and ecosystem functioning for a number of ecosystems (Loreau et al. 2001; Hooper et al. 2005, 2012; Cardinale et al. 2011). Two main candidate mechanisms have been proposed to explain positive biodiversity–ecosystem functioning relationships: selection and complementarity effects (Loreau et al. 2001). Selection effects occur when the most productive species are more likely to be included in species-rich communities and had greatest species-specific impacts on biomass (Fargione et al. 2007; Wang et al. 2013). Complementarity effects are thought to occur when species exhibit various forms of niche partitioning or facilitation that allow for a more complete use of resources in space or time and therefore larger influences on ecosystem functioning (Cardinale et al. 2007; Reich et al. 2012). Although most previous studies have mainly focused on taxonomic diversity (e.g., species richness) as measures of biodiversity related to ecosystem processes (Hooper et al. 2005), all of the mechanisms by which diversity is expected to affect ecosystem functioning strongly depend on the functional characteristics of local communities (McGill et al. 2006; Díaz et al. 2007; Hillebrand et al. 2008; Mokany et al. 2008; Roscher et al. 2012).

Functional trait composition of communities, manifesting the major aspects of biodiversity, is a key component that most often explains ecosystem functioning better than species richness per se (Díaz and Cabido 2001; Díaz et al. 2007; Flynn et al. 2011; Lavorel and Grigulis 2012; Roscher et al. 2013). Functional trait composition can be quantified by two main components: CWM (i.e., the average trait value of the species) and different indices of functional diversity (FD, i.e., the distribution of trait values representing the degree of overlap in trait values within the community) (Garnier et al. 2004; Mason et al. 2005). Ecosystem functioning is primarily determined by trait values of the dominant species to plant biomass, and thus can be predicted by CWM as suggested by mass ratio hypothesis (Grime 1998; Díaz et al. 2007; Roscher et al. 2012). Moreover, a larger functional dissimilarity among plant species is likely to increase the diversity in strategies of resource acquisition, which greatly promotes primary productivity as predicted by niche complementarity hypothesis (Díaz and Cabido 2001; Petchey and Gaston 2002, 2006). Despite the fact that the effects of FD on productivity were greatly dependent on the traits or niches considered, recent studies have reported that both components will be valuable predictors for primary productivity (Mouillot et al. 2011; Roscher et al. 2013). Overall, taxonomic diversity, functional identity, and functional diversity of plant communities are each known to determining community productivity, but their relative effects remain highly controversial (Hooper et al. 2005; Mouillot et al. 2011). Nevertheless, all these biodiversity components are not inherently exclusive and may simultaneously contribute to the variations in community productivity along environmental gradients (Mouillot et al. 2011).

In this study, we aimed to test the relative effects of taxonomic diversity, functional identity, and functional diversity on macrophytes community productivity along water depth gradient. First, we test how different measures of diversity and community productivity change along the gradient. Second, we examine whether the effects of functional trait composition on productivity are largely dependent on specific traits. Finally, we assess the relative importance of taxonomic diversity, functional identity (CWM), and functional diversity (FD) in determining the macrophytes community productivity along the gradient.

## Materials and Methods

### Field sampling designs

This study was carried out in Erhai Lake (25°52′N, 100°06′E) in Yunnan Province, China. In this Lake, macrophytes species showed a zonation distribution along water depth gradient. Most of macrophyte species can inhabit and dominate in the shallow water (e.g., 0–3.0 m depth), and only a few species can extent to deeper water. Macrophytes community composition and biomass were estimated in forty-two 25-m^2^ plots at seven sites of this lake (Fu et al. 2013). Sites were selected to represent the full range of water depth gradient and macrophytes community variation along this gradient. At each site, six 5 × 5 m plots were located along the water depth gradient in each 0.5-m interval as a depth stratus, extending from 0 m to 3.0 m water depth. The plots were randomly assigned to these six water depth gradients within macrophyte species dominated areas. Locations that were disturbed by recent human activity (e.g., mowing and fishing) were also excluded from sampling. Within each 25-m^2^ plot, there were three 0.2 m^2^ quadrats that we used for this analysis, as in these plots all species had been identified and recorded during fieldwork in October and September of 2011. Samples were collected using a rotatable reaping hook (diameter = 0.5 m, area = 0.2 m^2^), and it can usually uproot the vast majority of individuals within the quadrat in mud. The biomass of 17 macrophyte species within each of 126 quadrats was collected at each plot. Sampled plants were spun to remove excess water (about 3 min) and weighted to the nearest 0.10 kg fresh weight (FW). To measure the dry weight (g·m^−2^) of each species in each quadrat, we randomly collected 10–30 samples, weighted after oven-dried at 80°C for 48 h, and calculated the average wet per dry weight ratio for each species. The biomass of each species (community productivity) in each plot was calculated as the averaged biomass of each species and total biomass across the three quadrats within the 25-m^2^ plot, respectively.

### Functional traits measurements

We measured (or collected date from literature) 16 key functional traits on all 17 macrophyte species following standardized protocols. We sampled a total of 1513 individuals for the measurements on functional traits. The data of five functional traits (floating leaf, perennial, tuber, mean Julian flowering date, and flowering duration) were collected from regional floras.

Mean Julian flowering dates and flowering duration for each species were determined using regional floras that describe the earliest and latest months that a species is in flower. Information of species showing yes or no for three ordinal traits (i.e., floating leaf, perennial growth form, and tuber) was also obtained from the regional floras.

We measured the other 11 functional traits on 3–56 robust and healthy individuals of each species. Specific leaf area is part of the leaf economic spectrum and closely correlated with photosynthetic capacity, nitrogen content per mass, and leaf life span (Reich et al. 1999; Wright et al. 2004). Leaf dry mass content reflects the fundamental trade-off in investing resources in structural tissues vs. liquid-phase processes and therefore has been argued to be the root variable governing correlations among the traits in the leaf economic spectrum (Reich et al. 1999; Wright et al. 2004; Messier et al. 2010). Lamina thickness plays an important role in leaf and plant functioning and relates to species' strategies of resources acquisition and use (Kitajima and Poorter 2010). We collected a fully formed adult leaf, with no signs of damage or senescence at peak biomass. Collected leaves were stored in sealed plastic bags with a moist paper towel and scanned (to determine area) within 2 h of collection. Lamina thickness (mm) was measured on five to ten points per leaflets, avoiding the mid-vein. Leaves were then dried at 60**°**C for 4 days and weighed to determine leaf dry weight. Individual leaf area was calculated from the leaf scans using Image-Pro Plus (IPP) 6.0 (Media Cybernetics, Inc., Silver Spring, MD). Specific leaf area was calculated as leaf area (cm^2^) per unit of leaf dry mass (g), and leaf dry mass content as the ratio of a leaf dry mass to its water-saturated mass (g·g^−1^).

Leaf carbon [C] and nitrogen [N] were determined on 3–10 individual replicates per species. Samples were oven-dried at 80°C for 48 h and then ground to <0.5 mm using a Wiley Mill (Thompson Scientific, Swedesboro, NJ). Leaf carbon and nitrogen was analyzed on a Flash EA 1112 Elemental Analyzer (CE Elantech Inc., Lakewood, NJ) at Donghu Experimental Station of Lake Ecosystems, State Key Laboratory of Freshwater Ecology and Biotechnology, Institute of Hydrobiology, China. The leaf carbon/nitrogen [C/N] ratio was calculated as leaf [C] divided by leaf [N].

Ramet size (g) was calculated as the total dry weight (DW) of a single ramet. Shoot height (cm), a trait that is often allometrically related to overall plant size and competitive interactions for light (Westoby et al. 2002), was calculated as the distance from the basal stem to the top of photosynthetic tissues. Stem dry matter content is the dry mass-to-fresh mass ratio of twigs expressed as g·g^−1^. Stem diameter (mm) was measured as diameter at the basal stem (2–6 cm above the roots) using a vernier caliper, which is expressed as mm. Rooting depth (cm) was measured as the length of major roots for each species with expectation of *Ceratophyllum demersum*, because it is a rootless macrophyte species. This trait may be underestimated for submersed and floating-leaved macrophytes in that systematic sampling error did not allow us to sample absolutely intact roots of these species.

### Diversity and trait metrics

We selected a number of taxonomic and functional diversity indices with which to relate variations in ecosystem processes. For each plot, species richness, Simpson's evenness, and Simpson's diversity were calculated as measures of taxonomic diversity.

CWM trait values were used to describe the functional composition of each plot (Garnier et al. 2004) and were calculated as 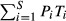, where *P*_*i*_ is the proportional biomass of *i*th species in the community, *T*_*i*_ is the trait values of species *i*, and *S* is the number of species. CWM trait values are a quantitative translation of the biomass ratio hypothesis (Grime 1998) and calculated as the sum across all species of the products of each species trait value and their relative abundance (Garnier et al. 2004).

FD indices based on single and multiple traits were used in the regression model to predict the community productivity. Functional trait diversity (FD_Q_) using Rao's quadratic entropy (Rao 1982) was computed as 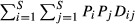, where *P*_*i*_ is the proportional biomass of *i*th species in the community, *D*_*ij*_ is the pair-wise trait dissimilarity of species *i* and *j*, and *S* is the number of species. Thus, FD_Q_ is the sum of the dissimilarities among all possible pairs of species in the trait space weighted by the product of species relative abundances. FD_Q_ based on single and multiple traits were calculated, respectively.

We also measured three functional diversity indices based on multiple traits: functional richness, functional evenness, and functional divergence (Villéger et al. 2008). Functional richness quantifies the convex hull volume of functional space occupied by the community, functional evenness represents the regularity of the distribution in abundance in this volume, and functional divergence represents the divergence in the distribution of the species traits within the trait volume occupied (Villéger et al. 2008; Spasojevic and Suding 2012). Functional evenness and functional divergence scale from 0 to 1; a high value indicates more regularity and more deviation, respectively, in the distribution of abundance of individuals in this volume (Villéger et al. 2008).

### Statistical analysis

A general linear mixed model was applied to assess the variations in community productivity, taxonomic diversity, functional identity (CWM), and functional diversity indices along the water depth gradient, with sites as random effects. The explanatory power of different sets of predictor variables and water depth gradient for variation in community biomass production was explored in a series of nested multiple regression model analysis (Roscher et al. 2013): (1) CWM trait values; (2) FD_Q_ based on single traits; (3) functional diversity based on multiple traits (functional richness, functional evenness, functional divergence, and RaoQ); and (4) species richness, species evenness, and Simpson's diversity. Firstly, we fitted linear regression models with each single predictor variable to evaluate their significance in explaining variation in community biomass. Secondly, models fitting initially all significant variables per predictor group as fixed effects were simplified through backward selection and step-wise exclusion of nonsignificant variables. Thirdly, the remaining candidate variables per predictor group were entered in a combined model including water depth gradient and its interaction with the predictor variables. The combined model was successively reduced by eliminating nonsignificant interaction terms first and nonsignificant main effects afterward.

## Results

### Taxonomic diversity along the gradient

**Figure 1:**
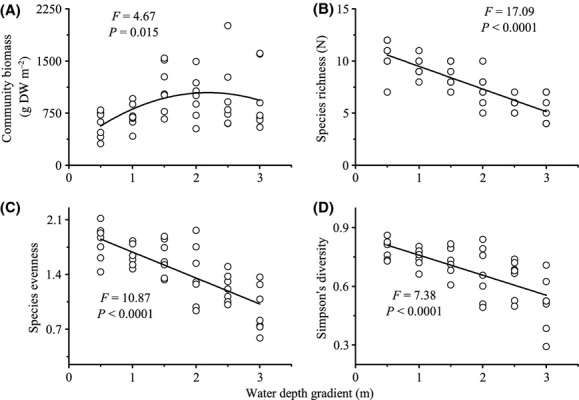
Relation between water depth gradient and (A) community biomass, (B) species richness, (C) species evenness, (D) Simpson's diversity. Open circles represent each of the 42 communities sampled. The regression line is drawn in black when it is significant.

Seventeen species including twelve submerged macrophytes, four floating-leaved macrophytes, and one floating macrophyte occurred in the 42 sampling plots across the water depth gradient in Erhai Lake (Table S1). The trait values of each species were showed in Table S1. Standing biomass of macrophyte communities increased from 310 g·DW·m^−2^ in the shallow water (0.5 m) to 2010 g·DW·m^−2^ in the deep water (2.5 m) (Fig. 1A). Overall, macrophytes community biomass showed a unimodel distribution along water depth gradient, with peaked biomass at intermediate depth (Fig. 1A). Species richness ranged from 4 to 12 species per plot with the median plot containing eight species. There were significant decreases in species richness, species evenness, and Simpson's diversity toward the deeper water (Fig. 1B,C and D), where a few dominant submersed macrophytes represented 60–80% of community biomass. Five perennial submersed macrophytes including three Potamogetonaceae species (*Potamogeton maackianus*, *Potamogeton lucens,* and *Potamogeton malaianus*) and two Hydrocharitaceae species (*Hydrilla verticillata* and *Vallisneria natans*) dominate across the water depth gradient, in which they represented >50% of the biomass.

### Functional identity and diversity along the gradient

**Figure 2:**
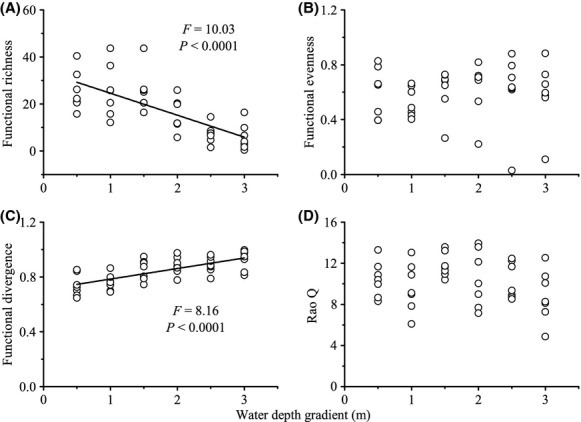
Relation between water depth gradient and multiple-trait functional diversity indices such as: (A) functional richness, (B) functional evenness, (C) functional divergence, (D) Rao Q. Open circles represent each of the 42 communities sampled. The regression line is drawn in black when it is significant.

Community-weighted means (CWM) showed significant decreases along the gradient for stem dry mass content, leaf [C], and leaf [N] (Table 1). Single-trait FD_Q_ exhibited significant decreases with increasing water depth for floating leaf, flowering duration, and rooting depth (Table 1). Functional richness decreased (Fig. 2A), while functional divergence increased significantly along the gradient (Fig. 2C). However, functional evenness and Rao Q indices did not change with varying water depths (Fig. 2B and D).

**Table 1:** Functional trait composition-environmental gradient–community biomass relationships. Functional trait composition is represented by components of community-weighted mean (CWM) traits and single-trait Rao Q diversity indices (FD_Q_). *F*-values were showed for specific traits showing significant regression relationships with environmental gradient or community biomass

Variable	Type of variable	CWM-Environment regression	CWM-Community biomass regression	FD_Q_-Environment regression	FD_Q_-Community biomass regression
Floating leaf	Ordinal: (1 = no, 2 = yes)		6.78* (+)	3.92** (−)	4.47* (+)
Perennial growth form	Ordinal: (1 = no, 2 = yes)		8.76** (−)		7.42** (+)
Tuber	Ordinal: (1 = no, 2 = yes)		5.58* (−)		5.01* (+)
Mean Julian flowering Date	Continuous (day)				5.60* (+)
Flowering duration	Continuous (Julian day)			2.56* (−)	
Ramet size	Continuous (mg)				12.51** (+)
Shoot height	Continuous (cm)		19.8*** (+)		22.86*** (−)
Stem diameter	Continuous (mm)		5.26* (−)		
Specific leaf area	Continuous (m^2^·kg^−1^)				
Leaf dry mass content	Continuous (g·g^−1^)		5.50* (−)		
Lamina thickness	Continuous (mm)				4.43* (−)
Rooting depth	Continuous (cm)			4.45** (−)	
Stem dry mass content	Continuous (g·g^−1^)	8.12*** (−)	4.44* (−)		
Leaf carbon content	Continuous (mg·g^−1^)	7.55*** (−)			17.8** (+)
Leaf nitrogen content	Continuous (mg·g^−1^)	3.62** (−)	4.59* (−)		19.4*** (+)
Leaf carbon/nitrogen ratio	Continuous (g·g^−1^)		8.94** (+)		

* *P* < 0.05;

** *P* < 0.01;

*** *P* < 0.001.

(+) indicates positive regression relationships; (−) indicates negative regression relationships.

### Predictions of community biomass

The variations in community biomass along the gradient were significantly dependent on CWM traits and single-trait FD_Q_. The CWM traits were positively related to community biomass for floating leaf, shoot height and leaf [C/N], and negatively for perennial growth form, tuber, stem diameter, leaf dry mass content, stem dry mass content, and leaf [N] (Table 1). Single-trait FD_Q_ were positively related to community biomass for floating leaf, perennial growth form, tuber, mean Julian flowering date, ramet size, leaf [C] and leaf [N], and negatively for shoot height and lamina thickness (Table 1). Multiple-trait FD were positively related to community biomass only for functional divergence (*R*^2^ = 0.15, *F* = 8.48, *P* = 0.006). Each individual group of predictor variables explained a significant proportion of total variation in community biomass (Table 2): CWM (57%), single-trait FD_Q_ (64%), multiple traits FD (26%), species richness (4%), water depth gradient (15%). The final model combining different groups of predictor variables explained over 65% of total variation in community biomass production (Table 2), in which each group of predictor contributed 61.5% for CWM, 30% for single-trait FD_Q,_ and 2.6% for water depth gradient.

**Table 2:** Multiple regression model analysis of different groups of predictor variables on community biomass production. The final model included the remaining significant variables per predictor group, water depth gradient, and its interaction as the predictor variables. The statistical parameters (i.e., *R*_Adj_^2^, df, *F*, *P*) were showed. FD indicates functional diversity, CWM indicates community-weighted mean, and FD_Q_ indicates Rao Q diversity indices

Group of predictors	*R*_Adj_^2^	df	*F*	*P*
Water depth gradient	0.15	2,39	4.67	0.015
Species richness	0.04	1,40	2.78	0.103
Species evenness	0.05	1,40	3.18	0.082
Simpson's diversity	0.03	1,40	2.23	0.143
Multiple-trait FD	0.26	2,39	8.21	0.001
Single-trait CWM	0.57	3,38	18.80	<0.0001
Single-trait FD_Q_	0.64	4,37	18.90	<0.0001
Final model	0.65	15,26	6.18	<0.0001

## Discussions

In this study, we applied a step-wise modeling procedure to test the relative effects of taxonomic diversity, functional identity, and functional diversity on macrophytes community productivity along water depth gradient and explore whether there were significant differences among different functional traits in explaining productivity. Study results showed that functional trait composition (i.e., functional identity and functional diversity) provided a more appropriate framework for explaining macrophytes community productivity in comparison with the taxonomic diversity. CWM exhibited a two times higher explanatory power relative to FD indices in determining variations in community productivity, supporting the mass ratio hypothesis that it is the traits of the dominant species which largely contribute to high productivity. Our results are consistent with previous study indicating a higher explanatory power of CWM relative to FD indices in determining variations in community productivity (Díaz et al. 2007; Roscher et al. 2013). Notably, FD indices also explained remarkable variations in community productivity, and the combination of CWM and FD would largely promoted the explanatory power of regressions models (Flynn et al. 2011; Roscher et al. 2013). This result suggests the mass ratio hypothesis (Grime 1998) and niche complementary hypothesis are not mutually exclusive in explaining the biodiversity–ecosystem functioning relationships.

For nine of sixteen traits, CWM and FD_Q_ showed significant correlations with community productivity, although the strength and direction of those relations depended on traits considered. The effects of CWM and FD on productivity can operate independently on the different traits or simultaneously on the same traits. For instance, communities with lower trait values of stem diameter, leaf/stem dry mass content, and leaf [C/N] showed higher productivity, while those with great even distributions of mean Julian flowering date, lamina thickness, and leaf [C] also had higher productivity. This finding suggests that ecosystem functioning may be influenced either by some traits' values of dominant species or by the other traits' distributions in communities. In addition, both mean values and spread distribution of floating leaf positively affected community productivity. Our results implied that the variation in community productivity may result from either specific functional component in some functional axes or both functional components in other functional axes. For perennial growth form, tuber, and leaf [N], however, productivity decreased with their mean trait values but increased with their distribution in a community. That is, functional composition in a community affected productivity through either additive or opposite effects of CWM and FD, depending on the traits considered. Remarkably, the negative relationships between functional diversity and ecosystem processes are impressive but not often reported by previous studies. This result suggests that the complementary effects on ecosystem functioning may be greatly dependent on the particular functional traits (Mokany et al. 2008). For some functional traits, productivity may increase with functional diversity; while for other traits, the higher productivity is largely due to all species possessing a particular trait value. Overall, after accounting for all candidate significant indicators in the finer model, FD showed a greatly positive effect on productivity, which demonstrates that complementary as a mechanism affecting ecosystem functioning may be associating with a number of trade-offs among different functional niches.

In contrary to the great effects of single-trait FD_Q_ on productivity, the multiple-traits FD contributed to a very less variations in community productivity, although the multi-trait FD indices may reflect unique information of functional composition and predict ecosystem functioning in certain condition. These results lend further supports to the idea that single-trait functional indices outperform multiple-traits indices in predicting ecosystem functioning (Butterfield and Suding 2013). The limited predictive power of multiple-traits FD was largely due to that the different individual traits were included in a composite index, which may have irrelevant or opposing effects on productivity (Table2). Therefore, the mix of positive, negative, and independent effects incorporated into calculations of multiple traits FD likely result in the relative few influences on productivity.

In present study, however, taxonomic diversity (i.e., richness, evenness, Simpson's diversity) generally explained very little variation in macrophyte community productivity. The low predictive power of species richness on productivity was particularly impressive. Most empirical researches on biodiversity–ecosystem functioning relationships have focused on species richness as a core in understanding the number of species in a community may influence ecosystem process (Hooper et al. 2005; Maestre et al. 2012). The observed weak effects of species richness and evenness on productivity suggest that the number and abundance of species present in a community may have little direct impact on ecosystem processes, and those variations in the functional identity and functional diversity of the communities will be of far greater importance.

With increasing water depth, we observed a significant decrease in taxonomic diversity, functional identity (i.e., CWM of stem dry mass content, leaf [C], and leaf [N]), and functional diversity (i.e., FD_Q_ of floating leaf, mean Julian flowering date, and rooting depth). However, community productivity tended to increase along water depth gradient. In present study, the influences of water depth on taxonomic diversity and productivity may operate independently of each other, because there were no significant relationships between taxonomic diversity and productivity. The significant negative effects of water depth on functional compositions were only found on particular traits, which also showed either irrelevant or negative influences on productivity. This result indicates that water depth may affect ecosystem functioning indirectly through functional composition. For the multiple-trait FD indices, functional richness decreased while functional divergence increased with water depth gradient. Previous studies have identified that functional richness is often positively correlated with species richness (Petchey and Gaston 2006). Similarly, functional richness had no significant effects on productivity. However, functional divergence showed a positive influence on productivity, lending further supports to the idea that water depth had an indirectly effect on ecosystem functioning.

In conclusion, the variations in functional trait composition (i.e., CWM and FD) exhibited remarkably great power in predicting variations in community productivity along environmental gradients. Functional composition in a community affected productivity through either additive or opposite effects of CWM and FD, depending on the particular traits being considered. Our results suggested both mechanisms of biomass ratio and niche complementarity can operate simultaneously on variations in community productivity, and considering both CWM and FD would lead to a more profound understanding of traits–productivity relationships.

## Supporting Information

**Table S1.** The mean values of 16 traits for the 17 occurred macrophyte species in the studied areas.
