# Supplementary material for: Functional traits composition predict macrophytes community productivity along a water depth gradient in a freshwater lake
Source: Ecol Evol. 2014 Mar 26;4(9):1516–23. doi: 10.1002/ece3.1022 (PMC4063455; doi:10.1002/ece3.1022)
Supplement: Supplementary file 1 [file ece30004-1516-SD1.docx]

Table S1. The mean values of 16 traits for the 17 occurred macrophyte species in the studied areas. Leaf [C] indicates leaf carbon content; Leaf [N] indicates leaf nitrogen content; Leaf [C/N] indicates leaf carbon/nitrogen ratio. The types and units of traits were showed in Table 4. The values of three ordinal traits (i. e, floating leaf, perennial growth form and tuber) were 1 (no) and 2 (yes).

| Species | Floating leaf | Perennial growth form | Tuber | Mean Julian Flowering Date | Flowering duration | Ramet size | Shoot height | Stem diameter | Specific leaf area | Leaf dry mass content | Lamina thickness | Rooting depth | Stem dry mass content | Leaf [C] | Leaf [N] | Leaf [C/N] |
| --- | --- | --- | --- | --- | --- | --- | --- | --- | --- | --- | --- | --- | --- | --- | --- | --- |
| *Potamogeton praelongus* | 1 | 2 | 2 | 224.5 | 83 | 1.75 | 58.67 | 1.63 | 166.50 | 6.50 | 0.09 | 0.16 | 12.36 | 42.97 | 2.61 | 0.29 |
| *Potamogeton pectinatus* | 1 | 2 | 2 | 205 | 152 | 5.30 | 132.72 | 1.18 | 82.26 | 6.98 | 0.45 | 0.15 | 7.58 | 38.74 | 1.45 | 0.20 |
| *Potamogeton perfoliatus* | 1 | 2 | 2 | 207 | 168 | 7.27 | 116.91 | 2.46 | 163.49 | 5.58 | 0.12 | 0.14 | 11.45 | 39.58 | 2.27 | 0.29 |
| *Najas marina* | 1 | 1 | 1 | 287 | 80 | 4.26 | 45.60 | 1.64 | 90.52 | 16.56 | 0.42 | 0.75 | 19.25 | 37.05 | 1.82 | 0.52 |
| *Potamogeton lucens* | 2 | 2 | 2 | 220.5 | 129 | 14.10 | 163.82 | 2.41 | 95.06 | 5.12 | 0.12 | 0.25 | 7.02 | 40.28 | 1.83 | 0.17 |
| *Hydrilla verticillata* | 1 | 2 | 1 | 207 | 110 | 4.11 | 106.57 | 1.24 | 118.95 | 6.10 | 0.10 | 0.46 | 10.29 | 35.61 | 3.12 | 0.29 |
| *Myriophyllum spicatum* | 1 | 2 | 2 | 154.5 | 111 | 4.92 | 90.02 | 2.00 | 109.61 | 7.21 | 0.29 | 0.54 | 7.83 | 37.53 | 2.66 | 0.21 |
| *Potamogeton maackianus* | 1 | 2 | 1 | 168 | 140 | 2.53 | 150.75 | 0.94 | 121.53 | 4.31 | 0.15 | 0.36 | 5.07 | 33.40 | 1.68 | 0.15 |
| *Ceratophyllum demersum* | 1 | 2 | 1 | 210 | 88 | 11.23 | 95.54 | 1.05 | 83.93 | 12.29 | 0.51 |  | 9.84 | 37.33 | 3.57 | 0.26 |
| *Vallisneria natans* | 1 | 2 | 2 | 222.5 | 133 | 9.93 | 92.44 | 5.29 | 73.20 | 13.80 | 0.64 | 0.39 | 4.66 | 35.91 | 2.29 | 0.13 |
| *Polygonum amphibium L.* | 2 | 2 | 2 | 223 | 78 | 15.36 | 152.17 | 3.14 | 44.99 | 4.19 | 0.21 | 0.09 | 4.08 | 46.19 | 1.99 | 0.09 |
| *Trapa natans L.* | 2 | 1 | 2 | 232 | 86 | 10.99 | 147.69 | 2.68 | 30.76 | 4.24 | 0.41 | 0.47 | 6.34 | 44.65 | 3.42 | 0.14 |
| *Potamogeton malaianus* | 1 | 2 | 2 | 227.5 | 127 | 8.15 | 151.46 | 2.02 | 89.36 | 4.98 | 0.12 | 0.20 | 7.01 | 41.79 | 3.00 | 0.17 |
| *Potamogeton intortifolius* | 1 | 2 | 2 | 218.5 | 115 | 8.05 | 149.07 | 2.22 | 75.12 | 4.76 | 0.11 | 0.29 | 7.07 | 38.61 | 2.73 | 0.18 |
| *Hydrocharis dubia* | 2 | 1 | 1 | 247.5 | 67 | 1.23 | 105.26 | 2.19 | 51.94 | 9.43 | 0.46 | 0.47 | 20.89 | 42.49 | 2.99 | 0.49 |
| *Potamogeton distinctus* | 2 | 2 | 2 | 209 | 150 | 5.56 | 80.67 | 2.57 | 58.53 | 5.56 | 0.26 | 0.26 | 7.59 | 41.43 | 2.80 | 0.18 |
| *Nymphoides peltatum* | 2 | 2 | 1 | 207.5 | 139 | 3.12 | 111.96 | 1.61 | 46.40 | 6.95 | 0.47 | 1.11 | 6.67 | 45.10 | 3.00 | 0.15 |
